# Side effects by oral application of atmospheric pressure plasma on the mucosa in mice

**DOI:** 10.1371/journal.pone.0215099

**Published:** 2019-04-09

**Authors:** Lukasz Jablonowski, Thomas Kocher, Axel Schindler, Karolina Müller, Frank Dombrowski, Thomas von Woedtke, Thomas Arnold, Antje Lehmann, Stefan Rupf, Matthias Evert, Katja Evert

**Affiliations:** 1 Unit of Periodontology, Department of Restorative Dentistry, Periodontology, Endodontology, Preventive Dentistry and Pedodontics, Dental School, University Medicine Greifswald, Greifswald, Germany; 2 Leibniz Institute of Surface Engineering (IOM), Leipzig, Germany; 3 Piloto Consulting Ion Beam and Plasma Technologies, Grimma, Germany; 4 Center for Clinical Studies, University Hospital Regensburg, Regensburg, Germany; 5 Institute of Pathology, University Medicine Greifswald, Greifswald, Germany; 6 Leibniz Institute for Plasma Science and Technology e.V. (INP Greifswald), Greifswald, Germany; 7 Department of Hygiene and Environmental Medicine, University Medicine Greifswald, Greifswald, Germany; 8 Technical University Dresden, Dresden, Germany; 9 Clinic of Operative Dentistry, Periodontology and Preventive Dentistry, Saarland University, Homburg, Germany; 10 Institute of Pathology, University Regensburg, Regensburg, Germany; Klinikum der Johann Wolfgang Goethe-Universitat Frankfurt Klinik fur Nuklearmedizin, GERMANY

## Abstract

Cold atmospheric pressure plasma (CAP) has been investigated with promising results for peri-implant diseases treatment. However, prior to *in-vivo* applications of CAP sources in humans, short-term harmful mucosal damage or other unwanted side effects have to be reviewed. 180 male mice (B6C3F1) were divided into twelve treatment groups (n = 15). The right buccal cheek mucosa was treated with CAP. The first and second group each received continuous 10 sec irradiation with 2 different plasma sources (kINPen09, PS-MWM). The third group was treated with the kINPen09 for one minute. Control groups were treated with a corresponding dose of ultraviolet light for 8 seconds or 48 seconds and the other one was left untreated. The animals were weighed before and after treatment. The animals were sacrificed one day or one week after exposure. Stained tissue samples were histologically examined for tissue damage independently by two experienced pathologists. One day after CAP treatment histological analysis showed focal mucosal erosion with superficial ulceration and necrosis accompanied by a mild inflammatory reaction. One week after CAP treatment, the mucosal defects were completely re-epithelialized, associated with remnants of granulation tissue in the stroma irrespective of treatment duration. Furthermore, no cytological atypia was found and no severe weight loss occurred. The control groups did not show any alterations at all. CAP treatment led to a superficial mucosal damage that healed within few days. Nonetheless, further long-term experiments are necessary to exclude undesirable side effects after longer observation time. Particularly, potential carcinogenic effects must be ruled out prior to the application of CAP treatment in daily dental practice.

## Introduction

Potential dental therapeutic applications of cold atmospheric pressure plasma (CAP) are manifold [1,2], one of the most promising properties is its antimicrobial activity [3–7]. Another property of plasma is its ability to modify a hydrophobic surface into a superhydrophilic one, which stabilizes clots and supports early wound healing of implants [8–11]. Thus, CAP may offer a novel approach for the treatment of peri-implant diseases [6]. Recently, cold atmospheric-pressure plasma devices have been developed, which have a temperature range between 40°C and 50°C in the interaction zone.

CAP treatment effects are based upon: i. reactive oxygen and nitrogen species (ROS, RNS; i.e. ozone (O_3_), nitric oxide (NO) radicals, hydroxide (OH) radicals, and super oxide anion radicals), ii. electric field, iii. VUV and UV radiation, iv. IR radiation. The complex composition may cause diverse effects and side effects on human cells. Above all, ROS and RNS as well as radiation energy have the potential to induce cellular toxicity by affecting cellular macromolecules (i.e. lipids and proteins) and to cause DNA damage [12,13].

Therefore, before becoming a safe medicinal product, many questions regarding the tolerability of plasma treatment need to be experimentally addressed. The first important question that needs to be answered is the amount of potential direct damage to the tissue on which CAP is applied and whether putative damage can be adequately resolved by the organism.

Several *in-vitro* and *ex-vivo* studies did not show any mutagenic or genotoxic effect due to plasma treatment and suggest that a clinical application of an argon plasma jet may be feasible [13–18]. However, only a limited number of *in-vivo* investigations dealt with this topic. Lademann and colleagues investigated the potential risks of a plasma source on the skin by focusing on UV radiation, temperature rise and reactive species. They showed that UV radiation of the tested plasma device was lower than the erythema dose for sunburn on the skin and that a thermal tissue damage could be excluded [19,20]. Furthermore, they did not find any side effects after the treatment of chronic wounds [19,20]. With healthy volunteers, Daeschlein et al. have demonstrated a good tissue tolerability of different cold atmospheric plasma devices with no disturbance of the skin barrier or reduction of skin moisture [21]. Short plasma treatment (10 sec and 30 sec) supported tissue recovery of acute, artificial wounds on the short-term (10 days) with no long-term (1 year) precancerous skin effects [22]. Van der Linde et al. tested skin sensitization of plasma treatment in a standardized murine model and could not detect any abnormal proliferation activity in the local skin or lymph nodes [23]. In a one-year follow-up risk assessment study, the long-term side effects of repetitive plasma treatment over 14 consecutive days in a rodent full-thickness ear wound model were investigated. No adverse effects with regard to genotoxicity could be observed, neither locally nor systemically [24,25].

In contrast to skin, human oral mucosa has no keratinized squamous epithelium, which makes it potentially more sensitive to damage from CAP treatment. It is known that oral tissue is more susceptible to carcinogenic changes due to UV radiation than the skin [26,27]. Up to now, only one very small *in-vivo* pilot study with six rabbits has examined the oral mucosa response after plasma treatment [28]; on days one and five after treatment no severe mucous membrane irritation could be shown. However, the number of treated animals in this study was too low to draw any reasonable conclusion for the tolerance of plasma treatment. Therefore, we investigated tissue damage after CAP treatment on oral mucosa of a collection of mice by histological tissue analysis.

## Materials and methods

### Animals

6–8 weeks old male mice (B6C3F1, Charles River Laboratories, Sulzfeld, Germany), weighing 25–30 g, were examined in this study (n = 180). Mice were housed in type 3 macrolon cages. There was a 12/12-hour rhythm of light and darkness and constant access to water and pelletized food. The condition of the animals and cages were monitored several times a day. The study was approved by the Committee for Animal Research (Landesamt für Landwirtschaft, Lebensmittelsicherheit und Fischerei Rostock, AZ 7221.3-1-057/13) in full accordance with the German Animal Protection Law.

### Treatment groups

**Fig 1:**
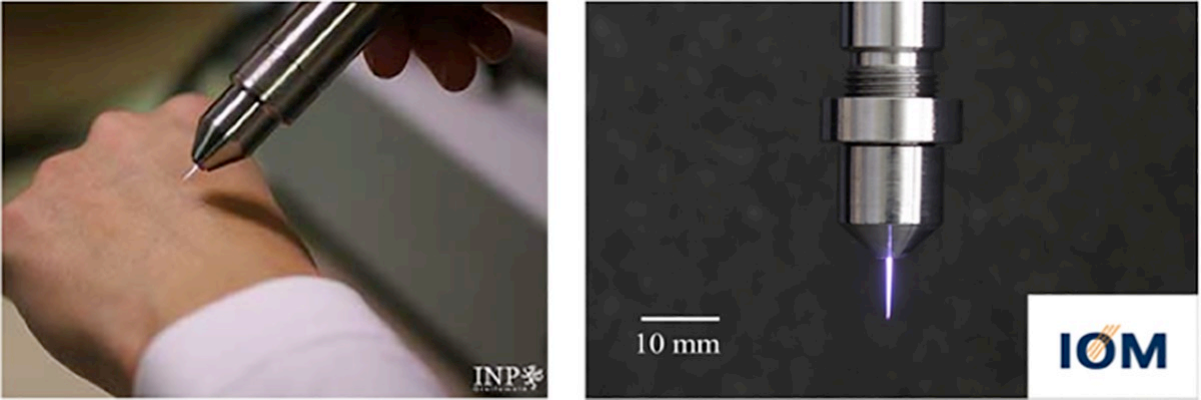
Plasma sources. Plasma source kINPen09 (INP, Greifswald, Germany) (left) and plasma source PS-MWM (IOM, Leipzig, Germany) (right).

Mice were divided into 12 groups of 15 animals each (Table 1). In the experimental groups they received a treatment with two different plasma sources: either kINPen09 or PS-MWM (Fig 1). The plasma source kINPen09 is an atmospheric-pressure plasma jet developed by the Leibniz Institute for Plasma Science and Technology Greifswald (INP) [29]. Argon (Air Liquide, Düsseldorf, Germany) was used as working gas with a flow rate of 5 standard liters per minute. The second plasma source (PS-MWM) was a microwave plasma source developed by the Leibniz Institute of Surface Engineering (IOM), Leipzig. The plasma source operated with helium and nitrogen (Air Liquide, Düsseldorf, Germany) with a total gas flow rate of 3.5 standard liter per minute [4,30]. Animals without any treatment or with ultraviolet (UV) treatment served as controls (Table 1).

**Table 1:** Experimental setup.

Animal group	Kind of treatment	Duration of treatment	Observation period		n
			1 day	1 week	
1	kINPen09	10 seconds	X		15
2	kINPen09	10 seconds		X	15
3	kINPen09	6 x 10 seconds	X		15
4	kINPen09	6 x 10 seconds		X	15
5	PS-MWM	10 seconds	X		15
6	PS-MWM	10 seconds		X	15
7	UV	8 seconds	X		15
8	UV	8 seconds		X	15
9	UV	48 seconds	X		15
10	UV	48 seconds		X	15
11	no treatment	-	X		15
12	no treatment	-		X	15

180 male B6C3F1-mice were divided into 12 groups: 6 experimental groups (1–6) and 6 control groups (7–12).

To simulate a single treatment session, the kINPen09 and PS-MWM exposure was carried out for 10 seconds (Table 1). In addition to examining the impact of a longer treatment, 6 x 10 seconds exposure was conducted with kINPen09, simulating a worst case scenario on one spot. The 60 seconds long treatment had to be interrupted because the mice needed rest intervals to breathe, otherwise they would suffocate from the Argon gas stream of the plasma.

Observation period in this experiment was one day and one week, respectively (Table 1).

### Experimental procedure

#### Anesthesia

All mice received a weight-adjusted intramuscular anaesthesia consisting of 50mg/kg ketamine and 10 mg/kg body weight xylazine (Selectavet Dr. Otto Fischer GmbH, Weyarn, Germany).

#### Plasma and UV treatment

**Fig 2:**
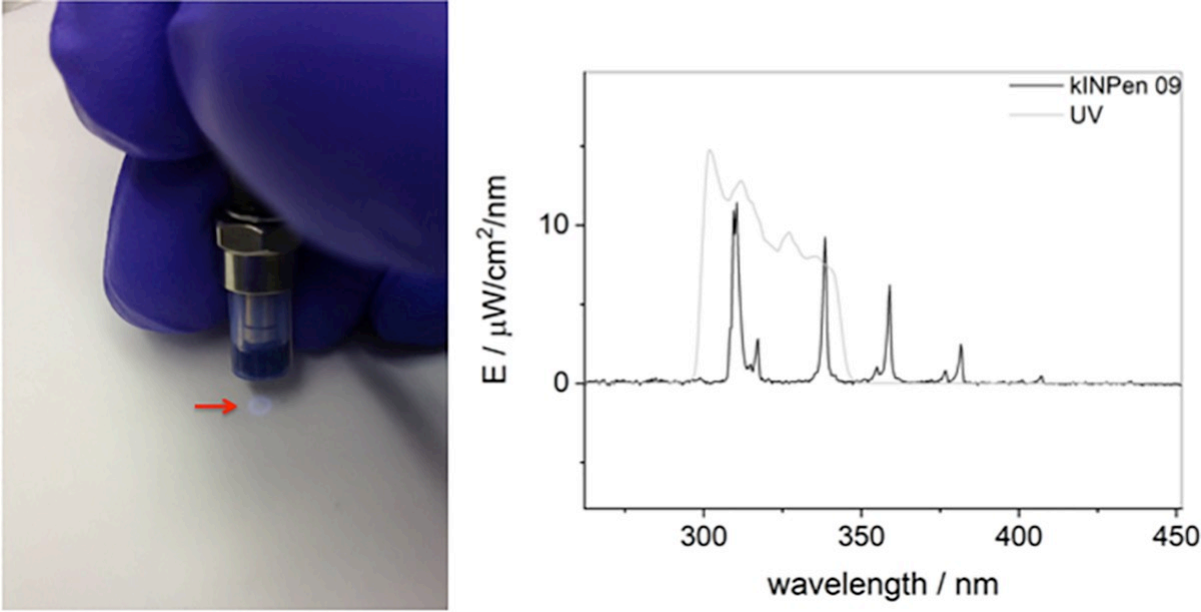
UV source. Xe flashlight with a power supply (Voltcraft Conrad Electronic, Wollerau, Switzerland) was used as UV source. Left: Visible UV emission at the end of the distance spacer (arrow). Right: Comparison of the UV spectrum of the kINPen09 (bold line spectrum) and the UV source (fine continuous spectrum).

**Fig 3:**
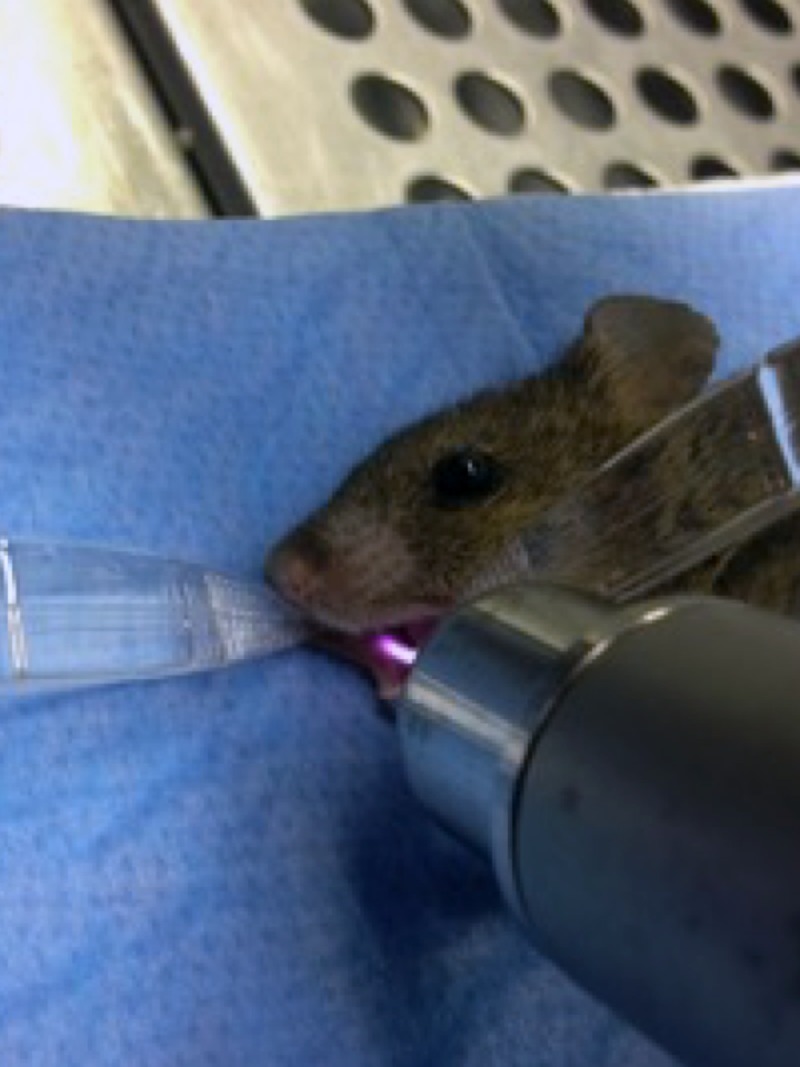
Plasma treatment (kINPen09). Treatment of the right cheek of a narcotized mouse.

The right cheek mucosa was kept open with anatomical plastic forceps (Mediware Servoprax, Wesel, Germany) and continuously treated with plasma for 10 seconds and 60 seconds, respectively, divided into six intervals of 10 seconds duration with 10 seconds resting time between the applications to give the animals the opportunity to breathe. A UV source (Xe flashlight with a power supply, Voltcraft Conrad Electronic, Wollerau, Switzerland, radiation energy of 155 μW/cm^2^) with a comparable UV spectrum to the kINPen09 (radiation energy of UVA/B 119 μW/cm^2^) was used to estimate a possible carcinogenic effect of UV emission (Fig 2). The corresponding control group received an 8-seconds and 48-seconds UV irradiation analogous (comparable radiation energy dose) to the kINPen09 plasma groups. It was ensured that the plasma/UV plume was in direct contact with the mucosa (Fig 3). The distance between the outlet of the device and the mucosa was between 7 to 10 mm. The contralateral left side was not treated and served as an intra-individual control.

#### Animal and tissue examination

Weight controls were performed before treatment as well as before animal sacrifice to assess the weight development. After the observation period, the mice were sacrificed by cervical dislocation. Immediately after, the cheeks were dissected, the oral mucosa was examined macroscopically, and tissue samples from the treated and the untreated mucosa were removed. After fixation in buffered formalin for 24 h, the samples were dehydrated by standard techniques and embedded in paraffin. Afterwards, 4 μm thick slides were cut with a microtome and stained with haemytoxylin and eosin (HE) and the Periodic acid-Schiff (PAS) reaction. Two experienced pathologists analyzed the slides microscopically (Nikon eclipse ci-L, Nikon, Tokyo, Japan) independently of each other and scored whether an inflammation, hyperplasia or dysplasia was present.

### Statistics

Descriptive analyses (frequencies [n], percentages [%], mean [m], 95% confidence interval [95% CI]) were used. The weight development was assessed as weight differences between baseline and day one or one week. T-test for unpaired samples was conducted to examine whether the observation period had an impact on weight development. For each observation period, an ANOVA was used to compare the weight differences between the experimental groups. Moreover, differences in macroscopical and histological mucosa alterations, on day one as well as inflammation for each observation period between untreated mice (n = 15) and the plasma group (n = 44, PS-MWM and kINPen 09) were examined with Fisher exact test. Differences in histological mucosa alteration on day one between mice in plasma groups (PS-MWM vs. kINPen 09 10s vs. kINPen 09 6 x 10s) were assessed with Chi-square-test. The program IBM SPSS Statistics 25 was used for all statistical analyses. Statistical significance was defined as p_(two-sided)_ ≤ .05.

## Results and discussion

### Treatment tolerance

The narcosis was well tolerated by the mice. No unforeseen incidents occurred and all animals survived the anesthetic procedure. In the consecutive days, there were no severe anomalies or problems in food intake. All animals except of two in the kINPen09 group survived the entire observation period. For no apparent reasons one animal was found dead in the cage and the other was sacrificed because of severe inflammation of the fur.

### Weight development

**Fig 4:**
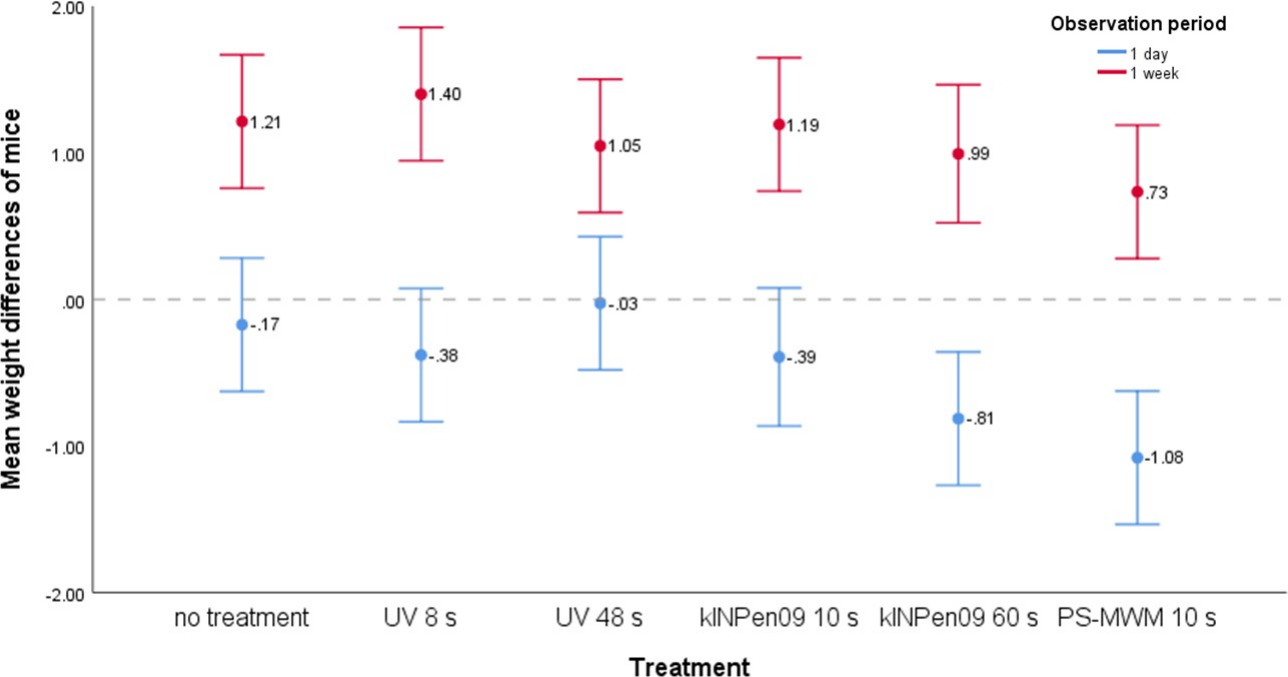
Body weight. Mean weight differences (95% CI) of mice broken down by observation period, treatment and treatment duration (N = 178). Weight decreased one day after treatment and weight increased one week after treatment. Mice receiving PS-MWM showed the highest weight loss and lowest weight gain. However, significant differences were only found after one day between mice receiving PS-MWM and mice without treatment (p = .013) as well mice receiving UV for 48 seconds (p = .002). The treatment duration had no impact on weight development.

The weight development was significantly associated with the observation period (t_(165.1)_ = -11.47, p < .001). Independent of treatment, on day one the mice showed a slight weight loss (m = -.48, 95% CI = -.65/-.31), while a weight gain was found on week one (m = 1.10, 95% CI = .88/1.31). Mice receiving a treatment with PS-MWM showed the greatest weight loss on day one and the lowest weight gain on week one (Fig 4). On day one, the mean weight difference differed significantly between treatments (F_(5, 83)_ = 4.55, p = .001) (Fig 4). Bonferroni corrected post-hoc tests showed that the weight development differed significantly between mice receiving PS-MWM and mice with no treatment (p = .013) as well as mice receiving UV for 48 seconds (p = .002). After one week, the mean weight differences were not statistically significant between treatments (Welch’s F_(5, 36.1)_ = 1.78, p = .142) (Fig 4). Treatment duration had no impact on weight development. Non-significant p-values are not reported.

### Effects of plasma on the mucosa

One day after treatment, the mucosa showed macroscopically mild changes in color from red to white in the treated area of all plasma treated groups (Table 2). After fixation in formalin these changes became weaker and were difficult to define macroscopically.

**Table 2:** Overview of mucosal alterations.

	Time of treatment	Time of observation	Number of mice	Mucosa alteration		Inflammation	Hyperplasia	Dysplasia
				Macroscopically	Histologically			
	1 day							
kINPen 09	10 s	1d	14	14 (100) a	8 (57) a	14 (100) a	0 (0)	0 (0)
kINPen 09	60 s	1d	15	15 (100) a	6 (40) b,c	15 (100) a	0 (0)	0 (0)
PS-MWM	10 s	1d	15	15 (100) a	13 (86) a,c	15 (100) a	0 (0)	0 (0)
UV	8 s	1d	15	0 (0)	0 (0)	0 (0)	0 (0)	0 (0)
UV	48 s	1d	15	0 (0)	0 (0)	0 (0)	0 (0)	0 (0)
Untreated	-	1d	15	0 (0) a	0 (0) a,b	0 (0) a	0 (0)	0 (0)
	1 week							
kINPen 09	10 s	1w	15	0 (0)	0 (0)	15 (100) a	0 (0)	0 (0)
kINPen 09	60 s	1w	14	0 (0)	0 (0)	14 (100) a	0 (0)	0 (0)
PS-MWM	10 s	1w	15	0 (0)	0 (0)	15 (100) a	0 (0)	0 (0)
UV	8 s	1w	15	0 (0)	0 (0)	0 (0)	0 (0)	0 (0)
UV	48 s	1w	15	0 (0)	0 (0)	0 (0)	0 (0)	0 (0)
Untreated	-	1w	15	0 (0)	0 (0)	0 (0) a	0 (0)	0 (0)

Number and percentage of a mice with mucosa alterations (macroscopically and histologically) in the different treatment and control groups stratified for observation one day and one week after treatment. (s = seconds, percentage in brackets). Fisher-exact-test: Non-significant p-values are not reported.

a: p<0.001 untreated vs. treatment group.

b: p = 0.017 untreated vs. treatment group.

c: p = 0.021 kINPen09 vs. PS-MWM.

Histologically, focal ulceration and necrosis with superficial homogenization of the underlying stroma and fibrin deposits accompanied by a mild inflammatory reaction were detected in all plasma treated groups one day after the treatment (Table 2). The inflammatory infiltrate consisted of neutrophil granulocytes and lymphocytes with a few plasma cells. Eosinophilic granulocytes were rarely detected.

**Fig 5:**
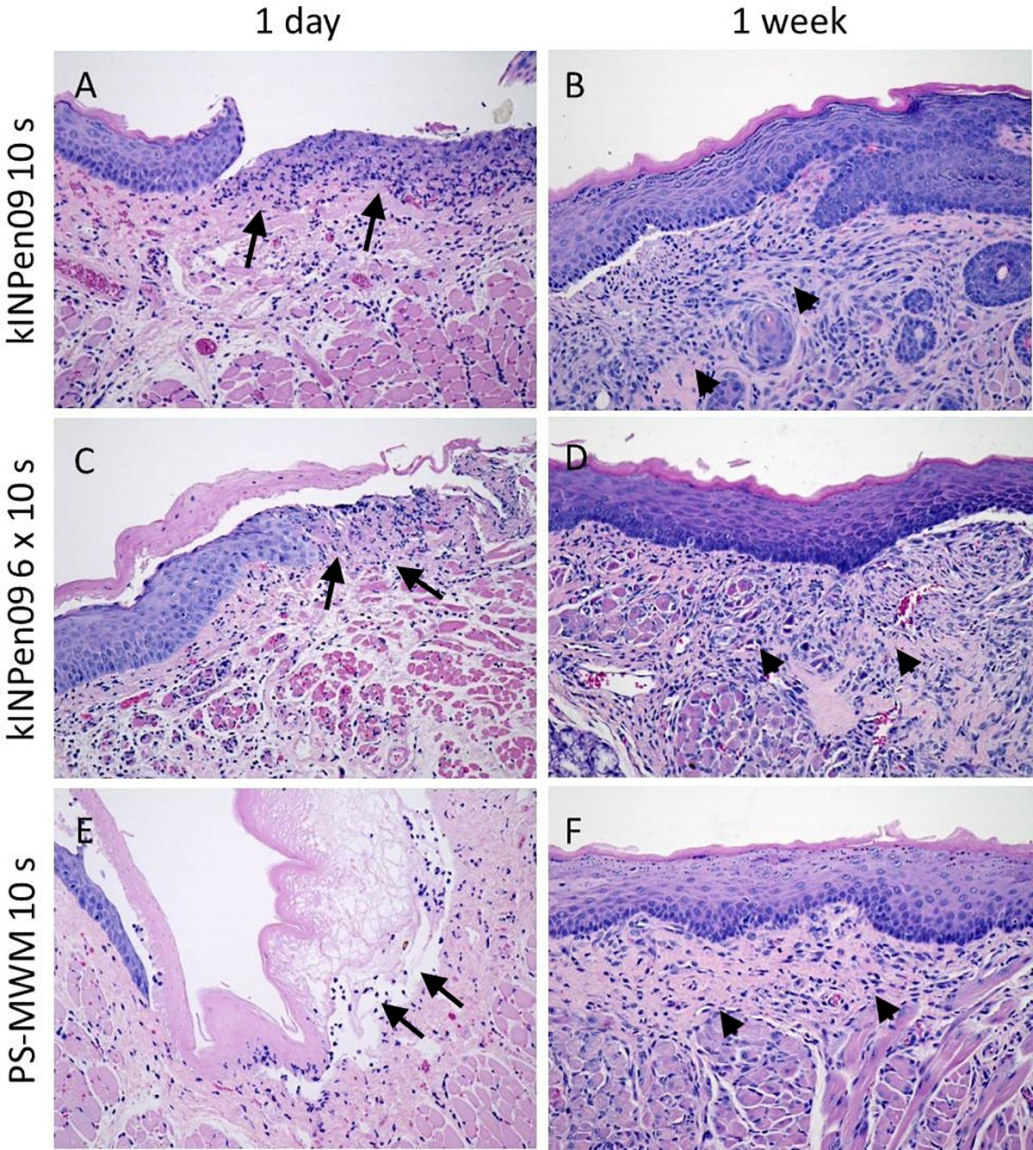
Histological examinations of plasma treated groups. A, C, E: Cheek mucosa with ulceration and inflammation in the stroma (arrows) one day after the treatment with CAP (A kINPen09 10s, C kINPen09 6x10s, E PS-MWM 10s). B, D, F: Cheek mucosa one week after the CAP treatment (B kINPen09 10s, D kINPen09 6x10s, E PS-MWM 10s) with reepithelialization and remnants of granulation tissue (arrow heads). (A-F: HE, original magnification 200-fold).

**Fig 6:**
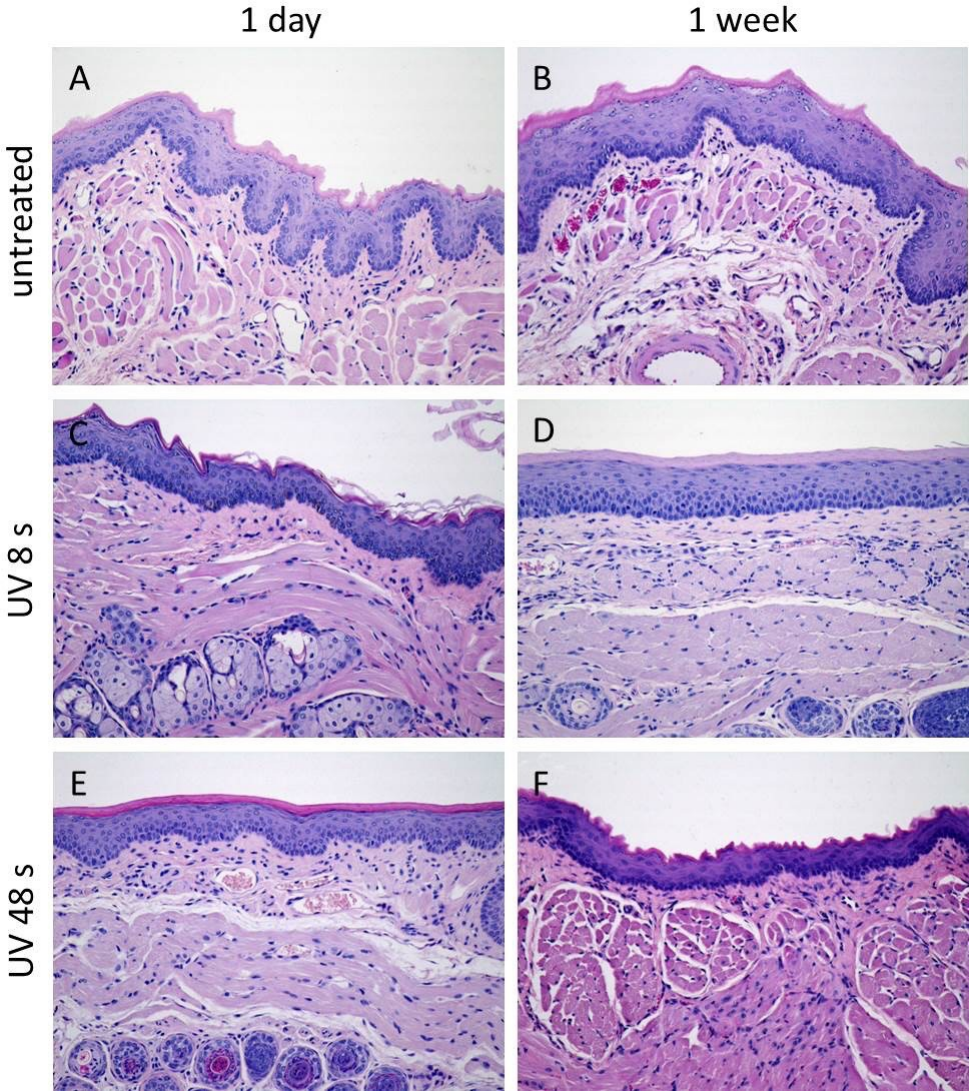
Histological examinations of control groups. A, B: Cheek mucosa of the untreated animals 1d (A) and 1 week (B) after the beginning of the experiments in the experimental groups. Mice cheek mucosa shows in contrast to human cheek mucosa slight keratinization. C, E: Cheek mucosa 1 day after UV-treatment with no alterations of the mucosa (C 8 s, E 48 s). D, F: Unaltered cheek mucosa one week after the UV-treatment (D 8 s, F 48 s). (A-F: HE, original magnification 200-fold).

Irrespective of the device used, one week after treatment, the mucosa did not show macroscopically any alteration in the region of the former treatment compared with the surrounding tissue. Histologically, the damaged epithelium of the oral mucosa was replaced by normal squamous epithelium, which was associated with remnants of granulation tissue in the stroma (Fig 5, Table 2). No granulomas could be observed. Furthermore, no cytological atypia was detected. Animals with UV and without any treatment showed normal organization of the mucosa (Fig 6, Table 2). The untreated left sided cheek mucosa was completely normal in all groups (S1 Fig).

Mice in control group (n = 90, untreated and UV) did not show any effects on mucosa alteration (macroscopically and histologically), inflammation, hyperplasia, and dysplasia, neither after one day nor after one week (Table 2). Inflammation could be detected in all mice receiving plasma (n = 88) one day and one week after treatment (Table 2). Moreover, one day after treatment, 100% of mice receiving plasma showed macroscopically mucosa alterations and 61% exhibited histological alterations of the mucosa (Table 2). However, one week after treatment, mice receiving plasma did not show mucosa alterations (Table 2). Macroscopic and histological mucosa alterations were most frequently detected in the group of mice receiving plasma after one day compared with mice in the control group (p < .001). Chi-square test showed that mice receiving plasma differed in histological mucosa alteration one day after treatment (χ^2^_(2, 44)_ = 7.04, p = .030). Post-hoc tests indicated that mice with PS-MWM had significantly more histological alterations than mice with kinPEN 09 for 60 seconds (p = .021). Non-significant p-values are not reported. As these tests were of explanatory character, no adjustment for multiple testing was performed.

Due to the absence of convincing studies on short-term side effects in the oral mucosa by CAP treatment, we sought to investigate the influence of two different plasma sources with different treatment times on the mouse oral mucosa. On day one, both plasma devices caused erosion and superficial ulceration with necrosis of the epithelial layer and inflammation in the underlying stroma. Irrespective of device and treatment time we found neither histological, nor cellular alterations one week after treatment.

The CAP-treatment itself led to slight macroscopic changes of the mucosa with only small mucosal defects in the treated area after day one. These mucosal defects were detectable macroscopically in all animals, whereas histologically, as a consequence of the small dimension of the lesions, we could only locate them in 40–80% of the mice (Table 2). Histological analyses showed, to a various extent, that CAP treatment physically destroyed the epithelial layer. In a previous study of our lab with a different CAP device with an Ar/O2 working gas etched a candida biofilm, and we concluded from these experiments that reactive oxygen species played a major role in biofilm removal and that plasma-assisted etching was mainly a chemically driven process, which was almost unnoticeable when using Ar without admixtures [31]. Presumably, the same reaction was responsible for epithelial tissue removal. Another obvious explanation may be that these damages are due to the increase in temperature, as the plasma treatment was performed stationary and with a very small distance. Even with cold plasma devices the temperature rises beyond 50°C if it is measured close within a 5 mm range [32]. Defects of the mucosa healed within one week, only histologically showing that the epithelium was regained, and remnants of granulation tissue were detectable without any signs of dysplasia. This feature corresponds to the proliferation stage of normal wound healing with a granulation tissue, which is comprised of collagen and extracellular matrix and into which a new network of blood vessels develops. In agreement with our previous candida treatment experiment, UV exposure did not ablate or abrade the thin epithelial layer [31].

One day after the treatment, all mice had a slight weight loss–including the control mice, which could be a consequence of stress due to anaesthesia. Mice receiving PS-MWM had the greatest weight loss and were significantly lighter than mice without treatment and with UV for 48s. This observation of weight loss goes along with mucosal damage in 13 of 15 PS-MWM animals, whereas in the kINPen09 group about 50% of the mice exhibited a mucosal defect. Epithelial abrasion causes pain, which prevented the animals from chewing. An analogous experience was reported by patients in whom a free gingival graft was harvested from the palate, as they reported pain and chewing difficulty [33]. One week after the treatment, all mice gained weight, and no significant differences in weight gain were found among the different groups at this timepoint. Thus, we conclude that all treatment procedures caused only, short-term constrains and a short period of pain.

We could not detect any difference between the 10 seconds and 60 seconds kINPen09 treatment, which is in agreement with *in-vitro* studies. Both Wende et al. and Kluge et al. did not find an increase in genotoxicity up to 180 seconds treatment with kINPen09 [13,17]. In addition, Kluge et al. investigated the chicken embryo viability as a further outcome where exposure times up to 2.5 min did not affect the viability [17]. The PS-MWM seems to have higher power efficiency with a higher temperature than the kINPen09, as we could detect more overt macroscopical and histological lesions and because the mice lost more weight. Since we do not have a direct comparison of the physical figures and properties of both devices, we do not have another explanation for these findings.

## Conclusions

In conclusion, the CAP-treatment is well tolerated in mice in short-term experiments. Further investigations are required to answer the most important question: How safe is atmospheric pressure plasma in long-term experiments? Is there a carcinogenic risk, in particular in cases of repetitive applications over a prolonged period or in subjects with additional risk factors for oral cancer such as smokers? To answer these questions, long-term experiments are necessary. Especially, a late manifesting co-carcinogenic effect of the intra-oral treatment must be ruled out before treatment with plasma can be established as an alternative therapy in dentistry.

## Supporting information

S1 Fig Histological examinations of untreated left sided cheek mucosa. The untreated left oral cheek mucosa did not show any alterations in all groups, here exemplary shown for the treatment with UV 48 s (A,B), kinPen09 6x10s (C,D) and PS-MWM 10s (E,F) (A—F HE, original magnification 200-fold, A,C,E one day after treatment, B,D,F one week after treatment).
(PDF)

## References

[1] Kim J-H, Lee M-A, Han G-J, Cho B-H. Plasma in dentistry: A review of basic concepts and applications in dentistry. Acta Odontol Scand. 2013; 72(1):1–12. 10.3109/00016357.2013.795660 24354926

[2] Cha S, Park YS. Plasma in dentistry. Clinical Plasma Medicine. 2014; 2(1):4–10. 10.1016/j.cpme.2014.04.002 27030818 PMC4808812

[3] Koban I, Matthes R, Hübner N-O, Welk A, Meisel P, Holtfreter B, et al Treatment of Candida albicans biofilms with low-temperature plasma induced by dielectric barrier discharge and atmospheric pressure plasma jet. New J Phys. 2010; 12(7):073039.

[4] Rupf S, Lehmann A, Hannig M, Schafer B, Schubert A, Feldmann U, et al Killing of adherent oral microbes by a non-thermal atmospheric plasma jet. Journal of Medical Microbiology. 2010; 59(2):206–12. 19910483 10.1099/jmm.0.013714-0

[5] Zhou X, Xiong Z, Cao Y, Lu X, Liu D. The antimicrobial activity of an atmospheric-pressure room-temperature plasma in a simulated root-canal model infected with Enterococcus Faecalis. IEEE Trans Plasma Sci. 2010; 38(12):3370–3374.

[6] Duske K, Jablonowski L, Koban I, Matthes R, Holtfreter B, Sckell A, et al Biomaterials. 2015; 52(C):327–34. 25818439 10.1016/j.biomaterials.2015.02.035

[7] Jablonowski L, Fricke K, Matthes R, Holtfreter B, Schlüter R, Woedtke T, et al Removal of naturally grown human biofilm with an atmospheric pressure plasma jet: An in‐vitro study. Journal of Biophotonics. 2016; 10(5):1–9. 10.1002/jbio.201600166 27539641

[8] Coelho PG, Giro G, Teixeira HS, Marin C, Witek L, Thompson VP, et al Argon-based atmospheric pressure plasma enhances early bone response to rough titanium surfaces. J Biomed Mater Res. 2012; 100(7):1901–6. 10.1002/jbm.a.34127 22492543

[9] Duske K, Koban I, Kindel E, Schröder K, Nebe B, Holtfreter B, et al Atmospheric plasma enhances wettability and cell spreading on dental implant metals. J Clin Periodontol. 2012; 39(4):400–7. 10.1111/j.1600-051X.2012.01853.x 22324415

[10] Canullo L, Penarrocha-Oltra D. Soft tissue cell adhesion to titanium abutments after different cleaning procedures: Preliminary results of a randomized clinical trial. Med Oral Patol Oral Cir Bucal. 2014; 19(2):177–183. 10.4317/medoral.19329 PMC4015045 24121917

[11] Choi S-H, Jeong W-S, Cha J-Y, Lee J-H, Yu H-S, Choi E-H, et al Time-dependent efects of ultraviolet and nonthermal atmospheric pressure plasma on the biological activity of titanium. Scientific Reports. 2016; 6:33421 10.1038/srep33421 27627871 PMC5024128

[12] Arjunan KP, Sharma VK, Ptasinska S. Effects of atmospheric pressure plasmas on isolated and cellular DNA-a review. Int J Mol Sci. 2015; 16(2):2971–3016. 10.3390/ijms16022971 25642755 PMC4346876

[13] Wende K, Bekeschus S, Schmidt A, Jatsch L, Hasse S, Weltmann KD, et al Risk assessment of a cold argon plasma jet in respect to its mutagenicity. Mutation Research—Genetic Toxicology and Environmental Mutagenesis. 2016; 798:48–54. 10.1016/j.mrgentox.2016.02.003 26994493

[14] Boxhammer V, Li Y-F, Köritzer J, Shimizu T, Maisch T, Thomas HM, et al Investigation of the Mutagenic Potential of Cold Atmospheric Plasma at Bactericidal Dosages. Mutation Research—Genetic Toxicology and Environmental Mutagenesis. 2013; 753(1):23–28. 10.1016/j.mrgentox.2012.12.015 23416235

[15] Isbary G, Koritzer JKX, Mitra A, Li Y-F, Shimizu T, Schroeder J, et al Clinical Plasma Medicine. Clinical Plasma Medicine. 2013; 1(1):36–44.

[16] Maisch T, Bosserhoff AK, Unger P, Heider J, Shimizu T, Zimmermann JL, et al Investigation of toxicity and mutagenicity of cold atmospheric argon plasma. Environ Mol Mutagen. 2017; 58(3):172–7. 10.1002/em.22086 28370324

[17] Kluge S, Bekeschus S, Bender C, Benkhai H, Sckell A, Below H, et al Investigating the Mutagenicity of a Cold Argon-Plasma Jet in an HET-MN Model. PLoS ONE. 2016;11(9). 10.1371/journal.pone.0160667 PMC5008819 27584003

[18] Bekeschus S, Clemen R, Metelmann H-R. Potentiating anti-tumor immunity with physical plasma. Clinical Plasma Medicine. 2018; 12:17–22.

[19] Lademann J, Richter H, Alborova A, Humme D, Patzelt A, Kramer A, et al Risk assessment of the application of a plasma jet in dermatology. J Biomed Opt. 2009;14(5):054025 10.1117/1.3247156 19895127

[20] Lademann J, Richter H, Schanzer S, Patzelt A. Comparison of the antiseptic efficacy of tissue-tolerable plasma and an octenidine hydrochloride-based wound antiseptic on human skin. Skin Pharmacology and Physiology. 2012; 25(2):100–106. 10.1159/000335558 22301799

[21] Daeschlein G, Scholz S, Ahmed R, Majumdar A, Woedtke von T, Haase H, et al Cold plasma is well-tolerated and does not disturb skin barrier or reduce skin moisture. Journal Der Deutschen Dermatologischen Gesellschaft. 2012 7;10(7):509–15. 10.1111/j.1610-0387.2012.07857.x 22405534

[22] Metelmann H-R, Vu TT, Do HT, Le TNB, Hoang THA, Phi TTT, et al Clinical Plasma Medicine. Clinical Plasma Medicine. 2013;1(1):30–5.

[23] van der Linde J, Liedtke KR, Matthes R, Kramer A, Heidecke C-D, Partecke L-I. Repeated Cold Atmospheric Plasma Application to Intact Skin Does Not Cause Sensitization in a Standardized Murine Model. Plasma Medicine. 2017; 7(4):383–93.

[24] Lloyd G, Friedman G, Jafri S, Schultz G, Fridman A, Harding K. Gas Plasma: Medical Uses and Developments in Wound Care. Laroussi M, Fridman A, editors. Plasma Process Polym. 2010; 7(3–4):194–211.

[25] Schmidt A, Woedtke TV, Stenzel J, Lindner T, Polei S, Vollmar B, et al One Year Follow-Up Risk Assessment in SKH-1 Mice and Wounds Treated with an Argon Plasma Jet. Int J Mol Sci. 2017;18(4):868. 10.3390/ijms18040868 PMC5412449 28422070

[26] Breger J, Baeva L, Agrawal A, Shindell E, Godar DE. UVB-Induced Inflammatory Cytokine Release, DNA Damage and Apoptosis of Human Oral Compared with Skin Tissue Equivalents. Photochemistry and Photobiology. 2013; 89(3):665–70. 10.1111/php.12030 23253030

[27] Agrawal A, Shindell E, Jordan F, Baeva L, Pfefer J, Godar DE. UV Radiation Increases Carcinogenic Risks for Oral Tissues Compared to Skin. Photochemistry and Photobiology. 2013; 89(5):1193–8. 10.1111/php.12140 23855371

[28] Liu D, Xiong Z, Du T, Zhou X, Cao Y, Lu X. Bacterial-killing effect of atmospheric pressure non-equilibrium plasma jet and oral mucosa response. J Huazhong Univ Sci Technol Med Sci. 2011; 31(6):852–6. 10.1007/s11596-011-0690-y 22173512

[29] Weltmann KD, Kindel E, Brandenburg R, Meyer C, Bussiahn R, Wilke C, et al Atmospheric Pressure Plasma Jet for Medical Therapy: Plasma Parameters and Risk Estimation. Contrib Plasma Phys. 2009; 49(9):631–40.

[30] Lehmann A, Pietag F, Arnold T. Clinical Plasma Medicine. Clinical Plasma Medicine. 2017; 7–8:16–23.

[31] Fricke K, Koban I, Tresp H, Jablonowski L, Schröder K, Kramer A, et al Atmospheric pressure plasma: a high-performance tool for the efficient removal of biofilms. PLoS ONE. 2012;7(8):e42539 10.1371/journal.pone.0042539 22880025 PMC3412829

[32] Bender C, Partecke L-I, Kindel E, Doering F, Lademann J, Heidecke C-D, et al The modified HET-CAM as a model for the assessment of the inflammatory response to tissue tolerable plasma. Toxicology in Vitro. 2011; 25(2):530–7. 10.1016/j.tiv.2010.11.012 21111803

[33] Zucchelli G, Mele M, Stefanini M, Mazzotti C, Marzadori M, Montebugnoli L, et al Patient morbidity and root coverage outcome after subepithelial connective tissue and de-epithelialized grafts: a comparative randomized-controlled clinical trial. J Clin Periodontol. 2010; 37(8):728–38. 10.1111/j.1600-051X.2010.01550.x 20590963

