# Supplementary material for: Side effects by oral application of atmospheric pressure plasma on the mucosa in mice
Source: PLoS One. 2019 Apr 9;14(4):e0215099. doi: 10.1371/journal.pone.0215099 (PMC6456225; doi:10.1371/journal.pone.0215099)
Supplement: S1 Fig — The untreated left oral cheek mucosa did not show any alterations in all groups, here exemplary shown for the treatment with UV 48 s (A,B), kinPen09 6x10s (C,D) and PS-MWM 10s (E,F) (A—F HE, original magnification 200-fold, A,C,E one day after treatment, B,D,F one week after treatment). (PDF) [file pone.0215099.s001.pdf]

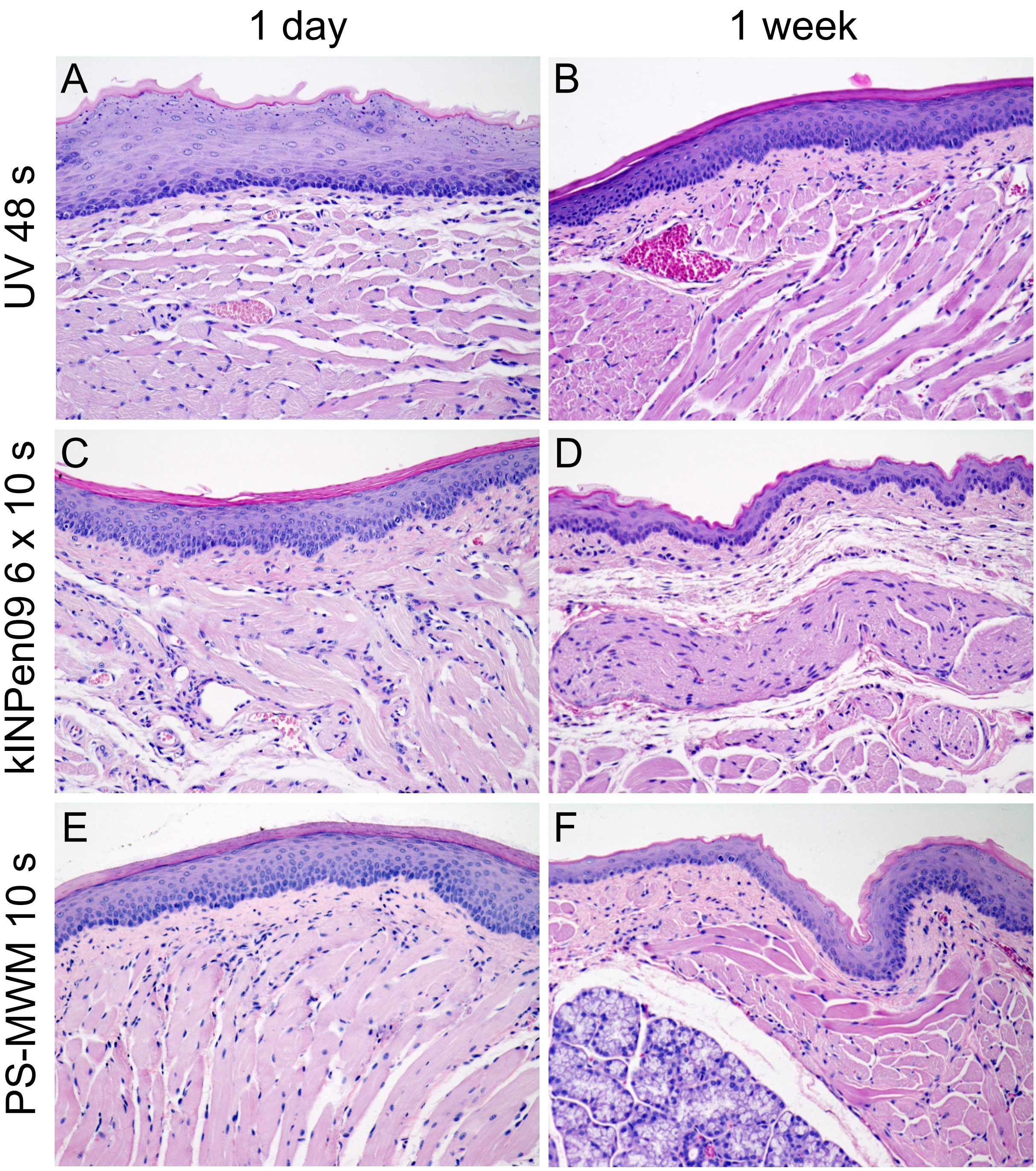

S1 Fig. Histological examinations of untreated left sided cheek mucosa. The untreated left oral cheek mucosa did not show any alterations in all groups, here exemplary shown for the treatment with UV 48 s (A,B), kinPen09 6x10s (C,D) and PS-MWM 10s (E,F) (A - F HE, original magnification 200-fold, A,C,E one day after treatment, B,D,F one week after treatment).
